# Cerebral Surgery

**Published:** 1890-08

**Authors:** 


CEREBRAL SURGERY.—Prominent among the names of those who, by careful investigation and bold operative interference, have done so much in recent times to develop cerebral surgery stands that of M. Lucas-Championnière, of Paris. To numerous previous papers advocating an extended practice of trephining in the treatment, both of injury and disease of the cranial contents, this surgeon has lately added a summary of a communication made to the Académie de Médecine, giving an instance of removal from the brain during life of an old clot, the result of spontaneous cerebral hemorrhage. The patient was a man, age 53, who, after an attack of apoplexy, had remained affected with paresis of the right lower limb, marked contraction of the right hand, and epileptiform attacks, which last, as time went on, increased more and more in frequency and intensity. Study of the symptoms presented in this case having led to the conclusion that they were due to a hemorrhagic deposit in the ascending frontal convolution, M. Lucas-Championniére decided, as the condition of the patient was in other respects good, on exposure of the supposed clot, with the view of liberating the compressed and irritated cerebral structures. After the skull had been trephined over the middle of the fissure of Rolando, an encysted clot was found embedded in the brain, and, as had been expected, near the middle of the convolution in front of this sulcus. The cyst having been freely opened, the rust-colored contents were removed, and the cavity washed out with antiseptic fluid.

The results of this operation, it is reported, were very satisfactory. The contraction of the right hand had ceased on the following day, and, when the patient was able to leave his bed, he found that he could walk with more ease. One attack of convulsions occurred about two months after the date of operation, but this was not repeated in the subsequent interval of four months, up to the time of the publication of this report. It is difficult at present, M. Lucas-Championniére states, to form any decided conclusions as to the prospects of trephining in cases of non-traumatic cerebral hemorrhage. Many cases, it is held, occur in which, after cerebral hemorrhage, compression and irritation play so direct a part as to lead to the conclusion that operative treatment may be often applied with success. M. Lucas-Championniére can fairly claim the merit of having done much to facilitate and improve the operative methods of cerebral surgery, and to show that trephining by itself is not attended with any great risk, and also that the indications for such procedure now extend over a wide field. In his latest contribution on this subject reference is made to as many as thirty cases of trephining in his own practice, performed with a view to the cure of intracranial affection, due to disease and not to injury, in all of which the patient recovered from the effects of the operation without the occurrence of any serious symptoms. Although in most of these cases, particularly in those of idiopathic epilepsy, the ulterior results of the operative treatment do not present instances of complete and brilliant success, they are still encouraging, and certainly justify the attempts that are being made to bring serious and very distressing affections of the brain within the range of surgical treatment.

—[*Editorial in British Medical Journal.*

